# Creating Alternative Afferent Input to Facilitate the Regeneration of Injured Primary Afferent Neurons

**DOI:** 10.7759/cureus.78708

**Published:** 2025-02-07

**Authors:** Emi Sawada, Toru Yamamoto, Naotaka Kishimoto, Dai Ooishi, Hiroyuki Sasakura, Kosei Takeuchi, Kenji Seo

**Affiliations:** 1 Division of Dental Anesthesiology, Faculty of Dentistry and Graduate School of Medical and Dental Sciences, Niigata University, Niigata, JPN; 2 Department of Medical Cell Biology, School of Medicine, Aichi Medical University, Nagakute, JPN; 3 Medical Research Creation Center, Aichi Medical University, Nagakute, JPN

**Keywords:** axon regeneration, nerve injury, sensory regeneration, synapse organizer, trigeminal nerve

## Abstract

The trigeminal spinal tract nucleus receives primary afferent input from the orofacial region, serving as a relay between peripheral terminals and secondary neurons. The trigeminal nerve is divided into ophthalmic, maxillary, and mandibular. While it is known that primary afferent terminals synapse with secondary neurons, the interaction between different primary terminals remains unclear. Recent studies have shown that trigeminal neurons with lost input can be activated through electrical stimulation of other afferent terminals. Therefore, we examined the possibility of inducing neural activity using synaptic organizers to promote circuit reorganization. To assess the regeneration of the injured inferior alveolar nerve (third division of the trigeminal nerve), the potential involvement of input from the infraorbital nerve (second division of the trigeminal nerve) in the regeneration of the injured inferior alveolar nerve (third division of the trigeminal nerve) was investigated. Intact and injured groups were created for the second and third divisions to facilitate comparative analysis. A synapse organizer was applied to establish input between the primary afferent terminals of these divisions. This study aimed to determine if central connections between different terminals can activate trigeminal neurons with lost input, ultimately promoting peripheral nerve regeneration. In this research, male C57BL/6J mice (seven to nine weeks old) (total n=40) underwent transection of the inferior alveolar nerve. They were divided into three groups: intact (n=10), injured (saline control) (n=10), and synapse organizer (n=10). In addition, the mice were divided into two groups: one group underwent inferior alveolar nerve transection only (II, intact; III, injured, n=5), and the other group underwent transection of both the infraorbital and inferior alveolar nerves (II, injured; III, injured, n=5), followed by local administration of a synapse organizer. Regeneration was assessed using immunostaining, sensory tests, and retrograde tracing. Regeneration was confirmed by retrograde tracing and functional recovery of sensory thresholds in the skin of the mental region. These findings align with previous observations that infraorbital nerve transection reduced regeneration activity, suggesting that infraorbital input triggered regeneration in the mandibular nerve. Thus, the results propose a novel therapeutic approach where mandibular nerve injury can be treated by stimulating the infraorbital nerve immediately after injury, enhancing peripheral nerve regeneration.

## Introduction

The injured peripheral nerve initiates axonal elongation from the injury site under Schwann cell guidance immediately after the injury [1-5]. Schwann cells recruit macrophages to remove debris from the site [6,7] and release regeneration-facilitating agents such as brain-derived neurotrophic factor and vascular endothelial growth factor [8-15], which result in axonal elongation. This sequence of actions can be targeted to enhance peripheral nerve regeneration after traumatic injury. An artificial nerve conduit connecting a transected nerve segment's proximal and distal ends can create a space for axon elongation while inhibiting the diffusion of facilitation factors [16-18]. However, the accumulation of excessive facilitation factors can eventually result in traumatic neuroma, which may cause dysesthesia.

Brief electrical stimulation after facial nerve injury potentiates the ability of the nerve to recover [19-22]. Such stimulation activates the injured nerve and can initiate the production of regeneration factors in the cell body [23,24]. Stimulating an injured trigeminal nerve to activate the cell body is very difficult. Suppose signal transmission between dendrites of different neurons can be achieved by mediating interneuron connections. In that case, artificial signal transmission might activate the injured cell, initiating the regeneration process in the injured neuron.

The spinal trigeminal nucleus receives primary afferent terminals from the orofacial region and relays their signals to secondary neurons [25]. The trigeminal nerve has three divisions (ophthalmic, maxillary, and mandibular), and their primary afferent terminals meet in sensory nuclei, which transmit signals to the brain [26]. It is well known that afferent terminals connect secondary neurons; however, the connections between different primary afferent terminals have not been well studied. It has been suggested that creating signal transmission between different divisions of the trigeminal nerve through electrical stimulation can activate neural regeneration in injured primary afferent nerve cells by stimulating neurons with lost input. Therefore, to assess the regeneration of the injured inferior alveolar nerve (third division of the trigeminal nerve), the potential involvement of input from the infraorbital nerve (second division of the trigeminal nerve) in the regeneration of the injured inferior alveolar nerve (third division of the trigeminal nerve) was investigated. Intact and injured groups were created for the second and third divisions to facilitate comparative analysis. A synapse organizer was applied to establish input between the primary afferent terminals of these divisions. Synapse organizers enable recovery from spinal cord injury and other neurological disorders through synaptic activation and connection [27]. Our study aimed to determine if central connections between different terminals can activate trigeminal neurons with lost input by stimulating afferent terminals, ultimately promoting peripheral nerve regeneration.

## Materials and methods

Animals

All experiments were approved by the Niigata University Intramural Animal Use and Care Committee (approval number #SA01047&A01640) and were performed in accordance with relevant guidelines and regulations. We followed the recommendations outlined in the ARRIVE guidelines for conducting research on animals. Animals were housed in an environment with controlled temperature (25°C) and humidity (approximately 40%), a 12-hour light/dark cycle, and free access to food and water.

A mandibular nerve transection model was used to investigate mandibular nerve regeneration. Both infraorbital nerve and mandibular nerve transection models were used to assess the impact of the infraorbital nerve on mandibular nerve regeneration.

Male C57BL/6J mice (seven to eight weeks) (The Jackson Laboratory Japan, Inc., Yokohama, Japan) were anesthetized using inhalational sevoflurane (Maruishi Pharmaceutical Co., Ltd., Osaka, Japan) and an intraperitoneal injection of a combination of medetomidine hydrochloride (Orion Pharma, Ltd., Espoo, Finland) (0.375 mg/kg), midazolam (Nichi-Iko Gifu Plant Co., Ltd., Toyama, Japan) (2 mg/kg), and butorphanol tartrate (Meiji Animal Health Co., Ltd., Kumamoto, Kumamoto) (2.5 mg/kg), with saline used as the solvent. Bilateral cheek incisions were made to expose the mandibular bone surface for bone cutting using a round bur; after exposing the inferior alveolar nerve, it was transected with microscissors and removed. After making a horizontal incision of approximately 5 mm on the maxillary buccal mucosa, the infraorbital nerve was exposed and transected, creating an infraorbital nerve transection model. Postoperative analgesia was provided using meloxicam (Boehringer Ingelheim, Ingelheim am Rhein, Germany) (0.1 mL/10 g). Mice were awakened using atipamezole hydrochloride (Orion Pharma, Ltd., Espoo, Finland) (0.1 mL/10 g).

The experimental groups were as follows: intact (no inferior alveolar nerve transection; n=10), injured (inferior alveolar nerve transection with saline injection into the medullary caudal nucleus; n=10), and synapse organizer (inferior alveolar nerve transection with substance synapse organizer injection into the medullary caudal nucleus; n=10) (Table 1).

**Table 1:** Number of animals in each group Intact: no inferior alveolar nerve transection, injured: inferior alveolar nerve transection with saline injection into the medullary caudal nucleus, synapse organizer: inferior alveolar nerve transection with substance synapse organizer injection into the medullary caudal nucleus, (II, intact; III, injured): the infraorbital nerve was left intact, and only the inferior alveolar nerve was transected, followed by the local administration of a synapse organizer into the caudal medullary subnucleus, (II, injured; III, injured): both the infraorbital and inferior alveolar nerves were transected, followed by the local administration of a synapse organizer into the caudal medullary subnucleus.

Group name	Number of animals
Intact	10
Injured	10
Synapse organizer	10
(II, intact; III, injured)	5
(II, injured; III, injured)	5

In addition, another experimental group was also established. The infraorbital nerve, corresponding to the second branch of the trigeminal nerve, is referred to as "II." In contrast, the inferior alveolar nerve, corresponding to the third branch of the trigeminal nerve, is referred to as "III." The group in which the infraorbital nerve was left intact and only the inferior alveolar nerve was transected, followed by the local administration of a synapse organizer into the caudal medullary subnucleus, was designated as the (II, intact; III, injured) group (n=5) (Table 1). The group in which both the infraorbital and inferior alveolar nerves were transected, followed by the local administration of a synapse organizer into the caudal medullary subnucleus, was designated as the (II, injured; III, injured) group (n=5) (Table 1).

Expression and purification of the synapse organizer used were performed as previously described [27]. The detailed characteristics of the molecule are as follows: The synapse organizer, cerebellin, facilitates synapse formation by mediating the interaction between presynaptic neuroligin and GluA. It connects presynaptic neuroligin with postsynaptic delta-type glutamate receptors (GluD) to form synapses. GluD is present only in a subset of excitatory synapses, such as those in the cerebellum, and cannot be used in other neural circuits. The synthetic synapse organizer was expressed and synthesized as an artificial chimeric protein that links cerebellin with the synaptic binding domains of neuronal pentraxin, which binds to GluA in nearly all excitatory synapses. This protein can reorganize and connect pre- and postsynaptic sites [27].

Injection of synapse organizer into the spinal trigeminal nucleus caudalis

One microliter of physiological saline or 1 μL of synapse organizer (1 μg/μL) was administered to the caudal part of the spinal trigeminal nucleus using an electronic microinjector (BJ-110 BEX; Bex Corporation, Toyota City, Japan) and a glass pipette made with a micropipette puller (P-97IVF; Sutter Instrument Co., Novato, CA, USA).

Immunohistochemical investigation of nerve regeneration

Synaptic Changes in the Spinal Trigeminal Nucleus Caudalis After Inferior Alveolar Nerve Injury

Three days after surgery, perfusion fixation was performed under deep anesthesia with sevoflurane using 4% paraformaldehyde in 0.1 M phosphate-buffered saline (PBS; pH 7.4) for 10 minutes. The brainstem was immersed in 30% sucrose for 24 hours and frozen at −80°C. Cryosections were prepared at a thickness of 20 μm using a cryostat (Leica, Tokyo, Japan) and mounted onto glass slides. Immunofluorescence staining was conducted at a position 500 μm from the obex. For membrane permeabilization, sections were treated with 0.2% Triton X-100 (Thermo Fisher Scientific, Waltham, MA, USA) for 15 minutes. The sections were blocked with 1% normal goat serum for 30 minutes. The primary antibody, anti-VGLUT1 (1:500), was diluted in a blocking solution and incubated at 4°C for 24 hours. Sections were washed three times with PBS. The secondary antibody (Alexa488, 1:500 or 1:2000) was incubated at room temperature for one hour. Sections were washed three times with PBS. Finally, the sections were mounted with VECTASHIELD (Vector Laboratories, Newark, CA, USA). Analysis was performed using ImageJ software (National Institutes of Health, Bethesda, MD, USA) over an area of 20.3 mm².

Verification of the Synapse Organizer Injection Site

Three days after surgery, perfusion fixation was performed under sevoflurane anesthesia using 4% paraformaldehyde in 0.1M PBS (pH 7.4) for 10 minutes. The medulla was then immersed in 30% sucrose for 24 hours. The tissue was frozen at −80°C and sectioned at 20 μm thickness using a cryostat. Sections were mounted on glass slides. Immunofluorescence staining was conducted at a position 500 μm from the hinge. For membrane permeabilization, sections were treated with 0.2% Triton X-100 for 15 minutes. Frozen sections were blocked with 1% normal goat serum for 30 minutes. The primary antibodies used included anti-GluA4 (1:1000), a glutamate receptor marker, and anti-His (1:500), a marker for the administered synapse organizer. Incubation with primary antibodies was carried out at 4°C for 24 hours. Sections were washed three times with PBS. Secondary antibodies (Alexa488, Cy3, 1:500 or 1:2000) were applied and incubated at room temperature for one hour. Then, sections were washed three times with PBS and mounted with VECTASHIELD.

Regeneration at the Distal End of the Transected Inferior Alveolar Nerve

On days 3 and 7 after surgery, perfusion fixation was performed under deep anesthesia with sevoflurane using 4% paraformaldehyde in 0.1 M PBS (pH 7.4) for 10 minutes. The mandible was then excised from the mice. The distal end of the transected inferior alveolar nerve was examined. The inferior alveolar nerve was transected at the mental foramen. The tissue was immersed in 30% sucrose for 24 hours, then frozen at −80°C. Using a cryostat, 20 μm thick sections were prepared from 500 μm proximal to the mental foramen and mounted onto glass slides. The mandible was decalcified using hydrogen chloride (Fujifilm Wako, Richmond, VA, USA) for 48 hours, followed by neutralization with a 5% sodium sulfate solution for 24 hours. Afterward, it was washed with PBS. The tissue was then immersed in 30% sucrose for 24 hours, frozen at −80°C, and sectioned into 20 μm thick slices using a cryostat, then mounted on glass slides. For membrane permeabilization, sections were treated with 0.2% Triton X-100 for 15 minutes. The sections were blocked with 1% normal goat serum for 30 minutes. Primary antibodies (β-3 tubulin (1:500), stathmin-2 (1:2000), S100β (1:200)) diluted in blocking solution were used and incubated at 4°C for 24 hours. Sections were then washed three times with PBS. The secondary antibodies (Alexa488, Alexa594, 1:500, or 1:2000) were incubated at room temperature for one hour. Sections were washed three times with PBS and mounted with VECTASHIELD.

Evaluation of regeneration in damaged nerves

Retrograde Neural Tracing

To trace regenerating axons, 3 μL of 1'-dioctadecyl-3,3,3',3'-tetramethylindocarbocyanine perchlorate (DiI; Thermo Fisher Scientific) was injected into the inferior alveolar nerve after transection. Under general anesthesia, DiI was injected on day 5 into the subcutaneous tissue around the mental foramen using a Hamilton syringe. Two days later, perfusion fixation was performed under sevoflurane anesthesia using 4% paraformaldehyde in 0.1 M PBS (pH 7.4) for 10 minutes. The tissue was then immersed in 30% sucrose for 24 hours and frozen at −80°C. Cryosections were prepared at a thickness of 20 μm using a cryostat and mounted onto glass slides. Sections were immersed in PBS for 15 minutes and then mounted with VECTASHIELD.

Measuring Tactile Threshold Changes in the Skin of the Mental Region

Facial sensory testing in mice was conducted following the principle of blinding. The von Frey hair test (Stoelting, Wood Dale, IL, USA) was used for assessment. Under general anesthesia, the skin at the skin of the mental region and the transection site was carefully shaved. Mice were placed on a metal mesh and enclosed in a light-proof case (SHINFACTORY Corp., Fukuoka, Japan). After approximately 30 minutes of habituation, baseline sensory thresholds and normal sensory state were confirmed to establish a reference for accurately evaluating experimental effects. The initial filament applied to control mice was 0.008 g. Similarly, after habituation, the skin at the skin of the mental region innervated by the inferior alveolar nerve was probed vertically with filaments on postoperative days 1, 3, and 7. Each filament was applied at five-minute intervals. The head withdrawal threshold (HWT) was the lowest filament weight that induced escape behavior in at least 3 out of 5 mechanical stimuli. The HWT for each mouse was defined as the average of three measurements. The HWT was measured before and on days 1, 3, and 7 after inferior alveolar nerve transection for the intact and synapse organizer treatment groups. The HWT was also measured for the vehicle treatment group. All HWT measurements were performed in a blinded manner.

Statistical analysis

Statistical comparisons were made using GraphPad Prism 10 software (GraphPad, La Jolla, CA, USA). Comparisons between the two groups were performed using the t-test. Statistical significance was set at p<0.05. All data were expressed as mean values ± standard deviation (SD).

## Results

Synaptic changes within the caudal subnucleus of the spinal trigeminal nucleus after inferior alveolar nerve transection

**Figure 1:**
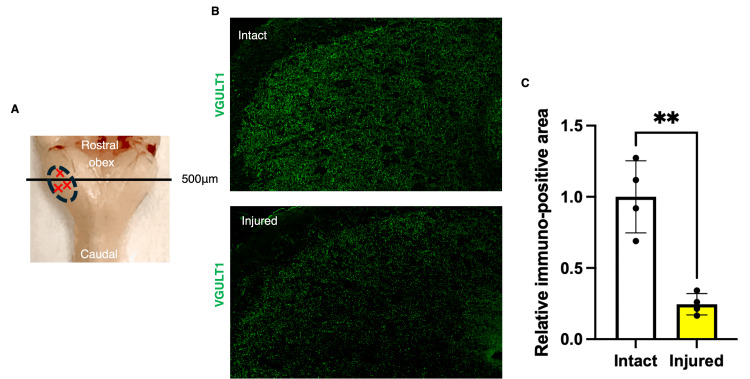
VGLUT1 in the caudal subnucleus of the trigeminal spinal nucleus (A) Cross-sectional preparation site of the mouse medulla oblongata. The black line indicates the position 500 μm from the obex. (B) Horizontal section of the caudal subnucleus of the medulla oblongata, showing immunofluorescence staining for VGLUT1. Synapse organizer or saline was injected on day 3 after surgery. (C) Comparison of VGLUT1 expression (**p<0.01, unpaired t-test, n=4, respectively). VGLUT1: vesicular glutamate transporter 1

The localization of vesicular glutamate transporter 1 (VGLUT1) in the caudal subnucleus of the trigeminal spinal nucleus was examined three days after surgery (Figure 1A). The study assessed how synaptic morphology changed after mandibular nerve transection. Immunohistochemical analysis using anti-VGLUT1 revealed that the area positive for VGLUT1 immunoreactivity was significantly lower in the injury group than in the intact group (p=0.0012, t-test; Figure 1B-1C).

Effect of synaptic organizer administration on axonal regeneration in the caudal subnucleus of the trigeminal spinal nucleus

Distribution and Co-localization of the Synapse Organizer in the Medulla and Receptors

**Figure 2:**
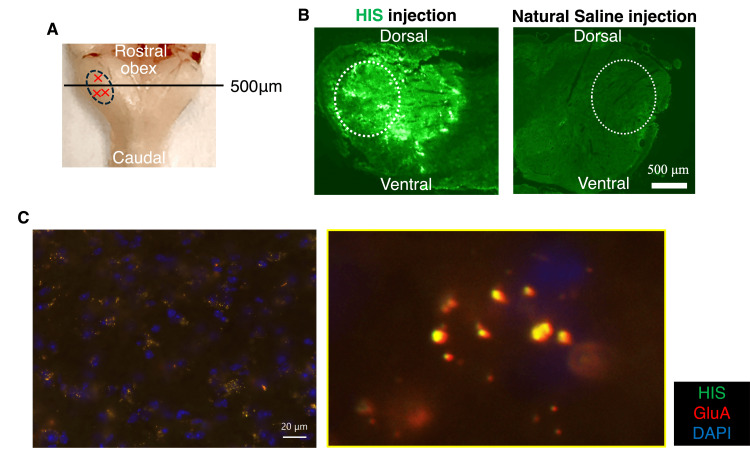
Co-localization of synapse organizer and GluA4 in the caudal subnucleus of the trigeminal spinal nucleus (A) Cross-sectional preparation site of the mouse medulla oblongata. The black line indicates the position 500 μm from the obex (B). Immunofluorescence staining of the medulla oblongata after local injection of synapse organizer – His-tagged or saline. The white dotted line marks the caudal subnucleus. The dotted line denotes the caudal subnucleus of the medulla oblongata. (C) Immunofluorescence staining showing co-localization of synapse organizer and GluA in the caudal subnucleus of the medulla oblongata. His: histidine

The signals exist at 500 μm from the foramen (Figure 2A). In the Vc region following injection of histidine (His)-tagged synapse organizer, a green fluorescent response was observed (Figure 2B). The His-tagged (green) and postsynaptic AMPA-type glutamate receptors (GluA) (red) in the trigeminal spinal subnucleus caudalis (Vc) appear in yellow dots, indicating His-tagged and GluA are co-localized in the same region (Figure 2C). This finding suggests that the administrated synapse organizer is retained in the Vc and combines with the presynaptic terminals labeled with His-tagged protein and postsynaptic GluA receptors in the medial terminals in the Vc.

Injection of the Synapse Organizer Promoted Regeneration of the Inferior Alveolar Nerve in the Inferior Alveolar Nerve Transection Model

**Figure 3:**
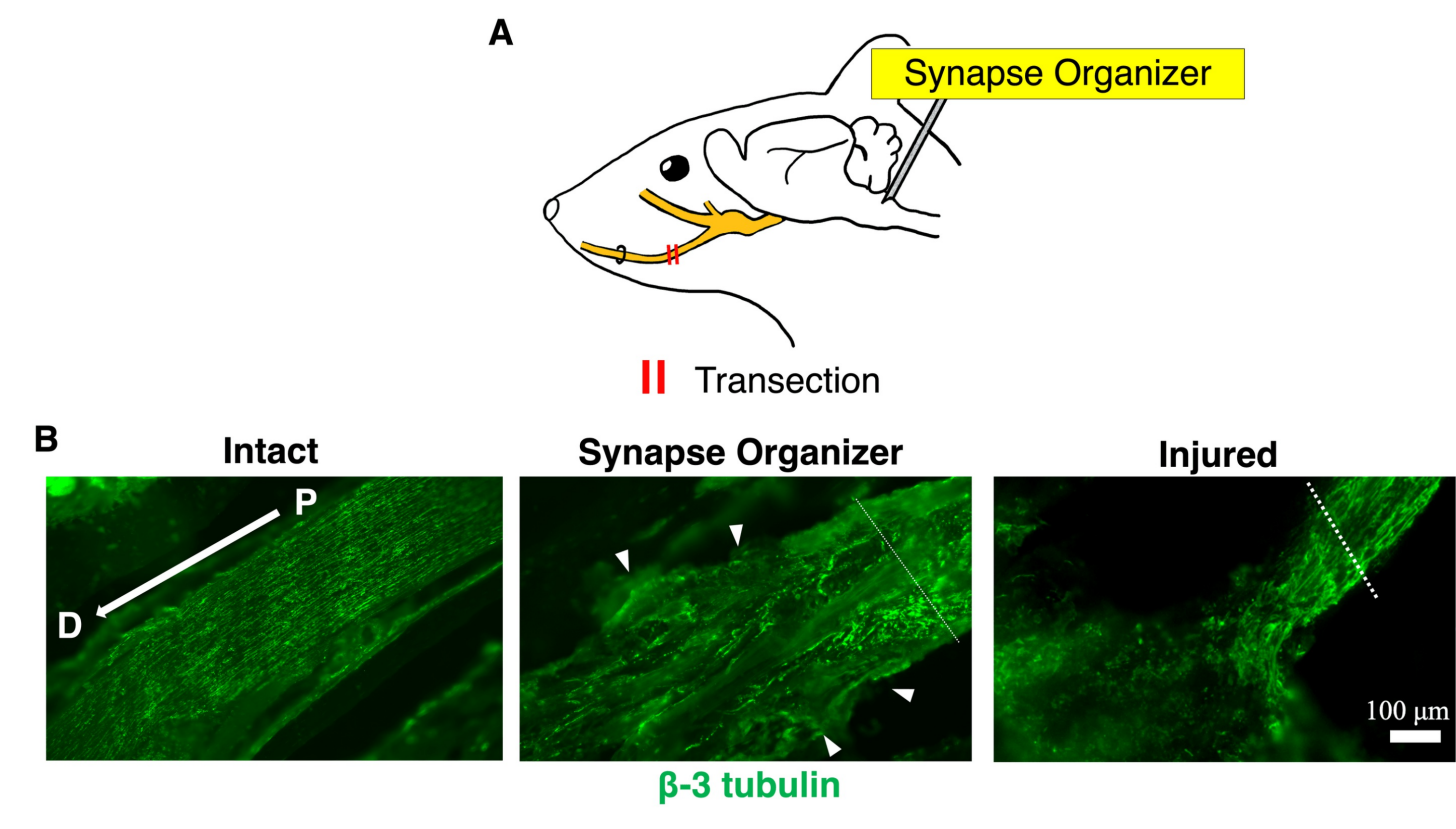
Expression of β-3 tubulin in the inferior alveolar nerve (A) Lateral view of a mouse head. (B) P: proximal side, D: distal side. Longitudinal section of the inferior alveolar nerve. Immunofluorescence staining of β-3 tubulin. D denotes distal and P denotes proximal. On day 3 after surgery, a synapse organizer or saline was injected into the caudal subnucleus of the medulla oblongata. The dotted line indicates the transection site. The inferior alveolar nerve with synapse organizer injection showed axonal growth. (A) Image Credit: Author

Regeneration of the inferior alveolar nerve was assessed three days after surgery. To examine how axons extend during regeneration of the transected inferior alveolar nerve, anti-β-3 tubulin was used to observe distal to the inferior alveolar nerve transection site in mice that underwent injection of synapse organizer into the Vc (Figure 3A). In the synapse organizer-treated mice, nerve fibers extended distally (Figure 3B). Conversely, nerve fibers extended in a radial and disorganized manner from the proximal end of the transection site in the inferior alveolar nerve of mice treated with saline (Figure 3B). This indicates that synapse organizer administration promoted nerve regeneration more effectively than saline.

Impact of infraorbital nerve injury on inferior alveolar nerve regeneration in synapse organizer injection model (infraorbital nerve and inferior alveolar nerve transection model)

**Figure 4:**
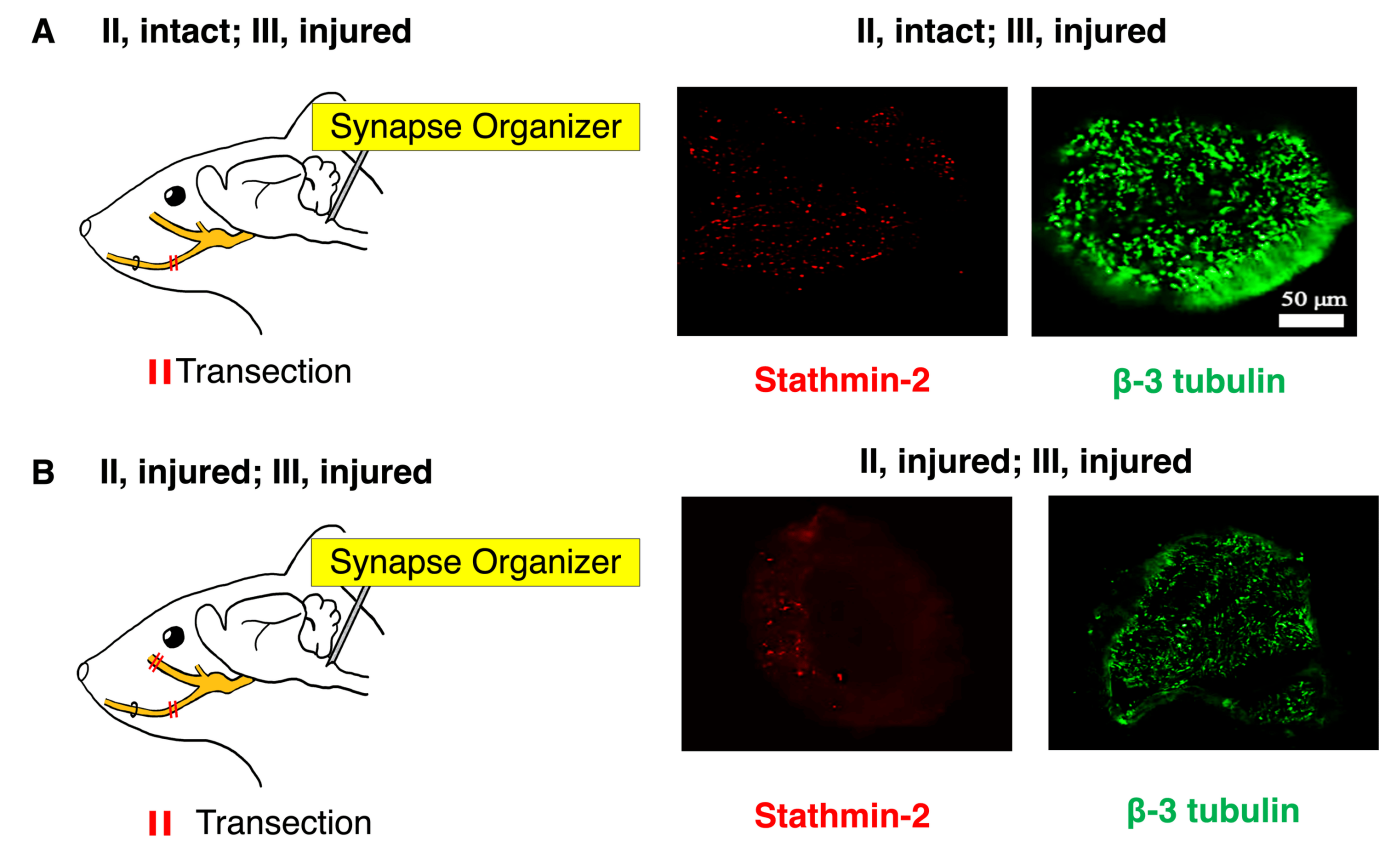
Expression of stathmin-2 and β-3 tubulin in the distal transection site of the inferior alveolar nerve (A) Lateral view of a mouse head. Transection of the inferior alveolar nerve with a synapse organizer administered to the caudal subnucleus of the medulla oblongata. Immunofluorescence staining of stathmin-2 and β-3 tubulin in a transverse section distal to the inferior alveolar nerve transection site three days after surgery. (B) Lateral view of a mouse head. Transection of the infraorbital and inferior alveolar nerves with a synapse organizer administered to the caudal subnucleus of the medulla oblongata. Immunofluorescence staining of stathmin-2 and β-3 tubulin in a transverse section distal to the inferior alveolar nerve transection site three days after surgery. (A-B) Image Credit: Author

Regeneration of the inferior alveolar nerve was assessed three days after surgery. To examine how axons extend during regeneration of the transected inferior alveolar nerve, anti-β-3 tubulin and anti-stathmin-2 were used. On day 3, cross-sectional analysis of the distal end of the transected inferior alveolar nerve showed that the expression levels of β-3 tubulin and stathmin-2 were higher in the (Ⅲ, injured; Ⅱ, intact) group (Figure 4A) than in the (Ⅲ, injured; Ⅱ, injured) group (Figure 4B). The inferior alveolar nerve showed better regeneration when the infraorbital nerve was preserved, and only the inferior alveolar nerve was transected.

**Figure 5:**
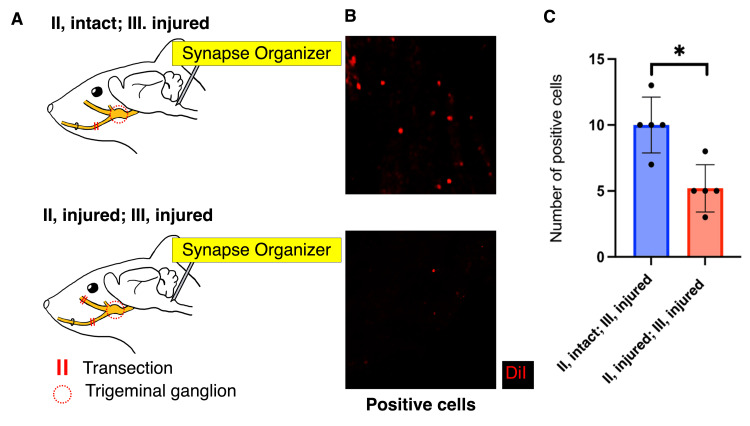
Retrograde tracing in the trigeminal ganglion (A) Lateral view of a mouse head. On day 7, a synapse organizer was injected into the caudal subnucleus of the medulla oblongata after surgery. (B) Immunofluorescence staining showed DiI-positive cells in the trigeminal ganglion. (C) Transverse section images for cases with transection of only the inferior alveolar nerve or the infraorbital and inferior alveolar nerves. Comparison of DiI-positive cell counts between (II, intact; III, injured) and (II, injured; III, injured) groups (*p<0.05, unpaired t-test, n=5, respectively). (A) Image Credit: Author

The number of DiI-positive cells in the trigeminal ganglion was significantly lower in the (Ⅲ, injured; Ⅱ, injured) group than in the (Ⅲ, injured; Ⅱ, intact) group (p=0.005, t-test; Figure 5). The red dye (DiI) injected near the mental foramen traveled through the connected segment of the transected nerve to the trigeminal ganglion, indicating that better nerve regeneration occurred when the infraorbital nerve was preserved and only the inferior alveolar nerve was transected.

**Figure 6:**
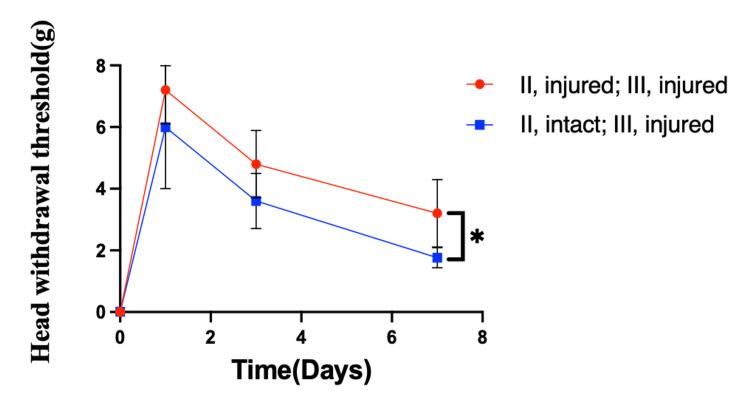
Sensory testing of the skin of the mental region using von Frey filaments On days 1 and 3 after surgery, the sensory threshold did not significantly differ between the (Ⅱ, intact; Ⅲ, injured) and (Ⅱ, injured; Ⅲ, injured) groups (p<0.05, unpaired t-test, n=5, respectively). On day 7, the (Ⅱ, injured; Ⅲ, injured) group exhibited a significantly higher sensory threshold (p<0.05, unpaired t-test, n=5, respectively).

On days 1 and 3 after surgery, the sensory threshold did not significantly differ between the (Ⅱ, intact; Ⅲ, injured) and (Ⅱ, injured; Ⅲ, injured) groups (p=0.14 on day 1 and 0.09 on day 3, t-test; Figure 6). On day 7, the (Ⅱ, injured; Ⅲ, injured) group exhibited a significantly higher sensory threshold (p=0.02, t-test; Figure 6). These results suggest that local administration of synapse organizer to the spinal trigeminal nucleus caudalis results in better regeneration of the transected inferior alveolar nerve when the infraorbital nerve is preserved.

## Discussion

The area of VGLUT1 immunoreactivity was significantly lower in the injury group than in the uninjured group, indicating that peripheral nerve transection reduces central input to second-order neurons. Additionally, the fluorescence immunoreactivity of β-tubulin and stathmin-2 was diminished after peripheral nerve transection, and the number of DiI-positive cells in the trigeminal ganglion was reduced. This suggests that the regeneration of injured primary afferent neurons was delayed. However, regeneration was activated by injecting an artificial synapse organizer at the relay site of the primary afferent nerve terminals. The injected synapse organizer was shown to co-localize with glutamate receptors in the medullary caudal subnucleus, the central site of primary afferent terminals of the trigeminal nerve. Disturbance of natural sensory input via another primary afferent induced delayed regeneration of the injured afferent nerve. These results suggest that an injured afferent nerve regeneration can be facilitated by adding central input to the injured nerve from another intact afferent nerve.

An artificial synapse organizer was administered to the trigeminal spinal nucleus caudalis, where nociceptive input from the orofacial region is focused and transmitted to secondary neurons. Primary afferent terminal dendrites of all three trigeminal nerve divisions are located close to this area. Our immunochemical study demonstrated that the administered artificial synapse organizer, attached to the presynaptic terminal, was close to postsynaptic GluA, suggesting newly created synaptic connections and signal transmission. Primary afferent terminals have been reported to express presynaptic AMPA receptors [28]. Therefore, an artificial synaptic organizer might connect dendrites in different primary afferent fibers, suggesting that infraorbital nerve afferent input is transmitted to mandibular nerve central terminals.

Regeneration activity of the injured primary nerve was estimated by increased immunostaining of stathmin-2 and β-3 tubulin in the injured nerve region. Regeneration was validated through the connection using the retrograde tracing method and functional recovery of the sensory threshold in the skin of the mental region. These results show that, in addition to the local administration of the artificial synapse organizer, the intact infraorbital nerve promotes regeneration activity in the inferior alveolar nerve. This suggests that afferent input via the infraorbital nerve triggered the induction of regeneration activity in the mandibular nerve. Such a mechanism might represent a new concept for the clinical treatment of mandibular nerve injury from the input of the infraorbital nerve. However, further research is needed before clinical application of artificial synapse formation in patients. The potential harmful effects of this treatment were not observed in mice. Although the trial in mice demonstrated a certain level of effectiveness, the response in humans may differ. This study proposes a novel treatment approach, and conducting future trials on human subjects will require more detailed and well-established research protocols. In the mouse trials, an extended follow-up period is planned to further evaluate the treatment's long-term effects, including potential adverse events or complications.

Limitations

Regeneration activity of the injured primary nerve was estimated by increased immunostaining of stathmin-2 and β-3 tubulin in the injured nerve region. Regeneration was validated through the connection using the retrograde tracing method and functional recovery of the sensory threshold in the skin of the mental region. These results show that, in addition to the local administration of the artificial synapse organizer, the intact infraorbital nerve promotes regeneration activity in the inferior alveolar nerve. This suggests that afferent input via the infraorbital nerve triggered the induction of regeneration activity in the mandibular nerve. Such a mechanism might represent a new concept for the clinical treatment of mandibular nerve injury from the input of the infraorbital nerve. However, further research is needed before clinical application of artificial synapse formation in patients. The potential harmful effects of this treatment were not observed in mice. Although the trial in mice demonstrated a certain level of effectiveness, the response in humans may differ. This study proposes a novel treatment approach, and conducting future trials on human subjects will require more detailed and well-established research protocols. In the mouse trials, an extended follow-up period is planned to further evaluate the treatment's long-term effects, including potential adverse events or complications.

## Conclusions

Novel synapse formation in the central nervous system induced by synapse organizer may facilitate the recovery of lost trigeminal sensory function. Furthermore, a novel therapeutic approach is proposed for mandibular nerve injury, where stimulation of the infraorbital nerve immediately after injury may potentially treat the condition and promote peripheral nerve regeneration. Future research is needed to explore the mechanisms further and evaluate the clinical applicability of synapse organizers in peripheral nerve sensory regeneration.
